# AP2/EREBP Pathway Plays an Important Role in Chaling Wild Rice Tolerance to Cold Stress

**DOI:** 10.3390/ijms241914441

**Published:** 2023-09-22

**Authors:** Songjin Yang, Jingming Zhou, Yaqi Li, Jiacheng Wu, Chuan Ma, Yulin Chen, Xingzhuo Sun, Lingli Wu, Xin Liang, Qiuping Fu, Zhengjun Xu, Lihua Li, Zhengjian Huang, Jianqing Zhu, Xiaomei Jia, Xiaoying Ye, Rongjun Chen

**Affiliations:** 1 State Key Laboratory of Crop Gene Exploration and Utilization in Southwest China, Rice Research Institute of Sichuan Agricultural University, Chengdu 611130, China; ysj_137@163.com (S.Y.); zhoujingming1992@163.com (J.Z.); liyq_106099@126.com (Y.L.); jiachengwu99@163.com (J.W.); 18981662375@163.com (C.M.); cchyulin@163.com (Y.C.); sxz15154123375@163.com (X.S.); m13627657830_1@163.com (L.W.); 15608188272@163.com (X.L.); d2229822870@163.com (Q.F.); 12541@sicau.edu.cn (Z.X.); lilihua1946@tom.com (L.L.); phosphate@126.com (Z.H.); 2 Demonstration Base for International Science & Technology Cooperation of Sichuan Province, Sichuan Agricultural University, Chengdu 611130, China; zhujianqing@163.com (J.Z.); jiaxiaomei@sicau.edu.cn (X.J.); 70166@sicau.edu.cn (X.Y.); 3 Crop Ecophysiology and Cultivation Key Laboratory of Sichuan Province, Rice Research Institute of Sichuan Agricultural University, Chengdu 611130, China

**Keywords:** cold tolerance, wild rice, AP2/EREBP, recombinant line

## Abstract

Cold stress is the main factor limiting rice production and distribution. Chaling wild rice can survive in cold winters. AP2/EREBP is a known transcription factor family associated with abiotic stress. We identified the members of the AP2/EREBP transcription factor family in rice, maize, and *Arabidopsis*, and conducted collinearity analysis and gene family analysis. We used Affymetrix array technology to analyze the expression of AP2/EREBP family genes in Chaling wild rice and cultivated rice cultivar Pei’ai64S, which is sensitive to cold. According to the GeneChip results, the expression levels of AP2/EREBP genes in Chaling wild rice were different from those in Pei’ai64S; and the increase rate of 36 AP2/EREBP genes in Chaling wild rice was higher than that in Pei’ai64S. Meanwhile, the MYC elements in cultivated rice and Chaling wild rice for the *Os01g49830*, *Os03g08470*, and *Os03g64260* genes had different promoter sequences, resulting in the high expression of these genes in Chaling wild rice under low-temperature conditions. Furthermore, we analyzed the upstream and downstream genes of the AP2/EREBP transcription factor family and studied the conservation of these genes. We found that the upstream transcription factors were more conserved, indicating that these upstream transcription factors may be more important in regulating cold stress. Meanwhile, we found the expression of AP2/EREBP pathway genes was significantly increased in recombinant inbred lines from Nipponbare crossing with Chaling wild rice, These results suggest that the AP2/EREBP signaling pathway plays an important role in Chaling wild rice tolerance to cold stress.

## 1. Introduction

Rice (*Oryza sativa* L.) is a very important crop, especially in developing countries. As a source of over one-third of the world’s population’s carbohydrates, it is widely cultivated on arable land. Rice can be divided into two main subspecies: japonica and indica [1]. Rice cultivation was originally achieved by sowing the annual indica subspecies [2]. A comprehensive map of the rice genome has shown that the japonica subspecies evolved via hybridization between early domesticated japonica rice and wild rice during the expansion process [3]. Low-temperature stress is a common abiotic stress that severely affects the growth and development of crops [4,5,6,7], ultimately impacting agricultural productivity and quality [8,9,10]. Low temperatures can affect all stages of rice, from the nutrient stage to the reproductive stage, during which the main phenotypes are greening, seedling stiffness and wilting, and death. During the nutrient growth stage, low-temperature stress may result in yellowness, reduced tillering, stiffness, wilting, or death of rice seedlings [11]. At the spike stage, low-temperature symptoms are characterized by branching degeneration, pollen reduction, spikelet abortion, and delayed spiking. Cold damage during the period from tetrad to early microspore [12] and cold damage at flowering stages may result in the arrest of anther development and insufficient fertilization [13]. Cold temperatures at the grain-filling stage affect photosynthesis and transport of photosynthetic products, which may lead to reduced grain fullness as well as rice quality [14,15]. In response to cold stress, plants undergo gene expression reprogramming, inducing or suppressing a series of functional and regulatory proteins [16,17]. Recent discoveries of two new low-temperature seedling survival rates (LTSS)-QTLs in rice provided an opportunity for marker-assisted breeding to enhance the cold tolerance of rice varieties and to genetically identify cold tolerance [18]. In maize and rice, calcium-dependent protein kinase (CDPK) can increase the expression of cold-regulated genes (*COR*) and C-repeat binding transcription factor (*CBF*) genes, thereby improving cold resistance by enhancing Ca^2+^ influx. Additionally, *OsCPK24* is a positive regulator of cold stress in rice, as it mediates calcium-dependent phosphorylation and inhibition of *OsGrx10* to maintain high levels of glutathione. Furthermore, *bZIP73*(*LOC_Os09g29820*) may contribute to the early adaptation of indica rice to cold climates during domestication. Understanding the molecular mechanisms of these genes and their regulatory pathways can provide new insights for the development of cold-tolerant crops [19,20].

**Figure 1:**
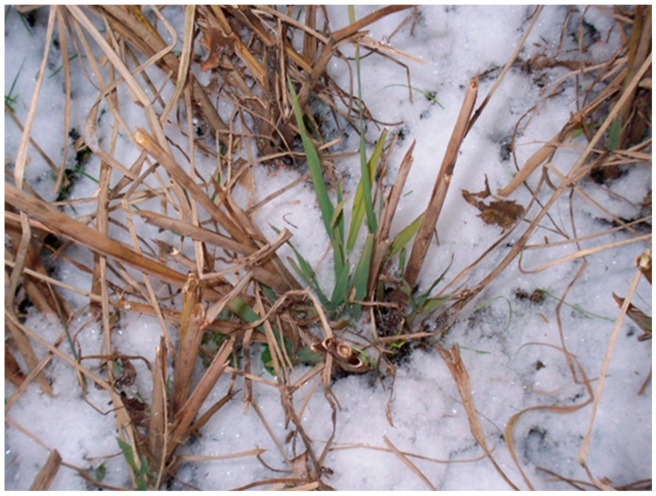
Chaling wild rice, unlike other varieties of rice, can survive in subzero conditions.

Due to its lengthy evolutionary history, wild rice has recently attracted researchers’ attention. Chaling wild rice, Dongxiang wild rice, and other wild rice can all survive with a 100% survival rate in winter at a temperature of −3 °C (Figure 1), while Pei’ai64, Nipponbare, 93-11, and Nanjing11 cannot survive [21]. It was found that rice mutant, Zixiangnuo early flowering mutant (ZXN-E), can affect the flowering period. And its missing 7 bp locus only exists in wild rice [22]. In wild rice from Malaysia, a gene encoding a silicon transporter protein (*Lsi1*) was identified that increased silicon uptake and accumulation, altered rice antioxidant activity, and morphological properties [23]. In India, introducing the *Os11Gsk* gene in wild rice into a good indica rice restorer line (KMR3) resulted in a significant increase in yield [24]. In Bangladesh, 24 rice varieties were compared so that the better phenotypes among them could be screened and documented in favor of parental selection for rice breeding [25]. Wild rice possesses superior genetic diversity compared to cultivated rice due to artificial preference selection during domestication. This abundant gene pool of wild rice can be utilized to improve the tolerance of cultivated rice to abiotic stress or diseases [26]. It was reported that wild rice (*Oryza rufipogon Griff*) is one of the cold-tolerant rice varieties, which is more resistant to low-temperature stress than indica rice varieties and can survive even at −1.0 °C [21] with much higher cold tolerance than generally cultivated rice. Under low-temperature conditions, the activity of superoxide dismutase (SOD), peroxidase (POD), catalase (CAT), ascorbic acid (AsA), and glutathione (GSH) in wild rice increased more significantly than in sensitive rice varieties [27,28]. Recent observations have shown that wild rice can survive cold winters and regenerate buds, indicating its potential as a hardy crop [29]. This opens up possibilities for identifying genes with different low-temperature expression patterns in wild rice and cultivated rice and using these genes to enhance the low-temperature tolerance of cultivated rice via transgenic methods.

Transcription factors (TFs) play a crucial role in regulating gene expression by binding to the promoter region of target genes [30,31,32,33]. Among various plant transcription factor families, the AP2/EREBP family is a significant group of transcription factors with diverse functions. This family comprises four major subfamilies, namely AP2, RAV, ERF, and DREB [34,35]. Under low-temperature stress, the C-repeat binding factor/dehydration-responsive element-binding 1(*CBF/DREB1*) genes are transiently induced, which further regulates downstream gene expression to cope with cold stress [36,37]. This DREB1/CBF cold response pathway is also conserved in rice, and *DREB1* genes have been found to enhance rice’s tolerance to environmental stress. Overexpression of *DREB1/CBF* genes has been reported to improve the cold resistance of *Arabidopsis*, *canola*, *tomato*, *tobacco*, and *rice*. Additionally, an inducer of CBF expression 1 (ICE1) or its homologs in plants [38,39,40,41,42] has been associated with increased expression levels of *CBF/DREB1* and *COR* genes, leading to improved freezing tolerance [43,44,45,46]. Studies have also shown that the ERF subfamily genes in rice are induced under low-temperature stress [35].

In this study, we identified AP2 gene families in rice, maize, and *Arabidopsis thaliana*, analyzed their characteristics, and investigated the expression of 135 AP2/EREBP genes at low temperatures using Affymetrix array technology. The results of the analysis showed that there were differences in the gene expression patterns of the AP2/EREBP family in Chaling wild rice and cultivated rice cultivar Pei’ai64S. The promoter sequences of the AP2/EREBP genes were analyzed to explore the potential genetic variants responsible for this differential expression. The results showed that the rice genes *Os01g49830*, *Os03g08470*, and *Os03g64260* have more MYC elements in wild rice. We also analyzed the upstream and downstream regulatory genes of the AP2/EREBP genes and found that the AP2/EREBP family of genes is conserved, suggesting that they play an important role in the functional expression of these genes. This study provides valuable insights into the genetic basis of cold tolerance in rice.

## 2. Results

### 2.1. Identification of Genes for AP2 Family Proteins in Rice

A total of 120 AP2/EREBP family genes were obtained from the Rice Genome Annotation Project (RGAP) and GRAMENE and were listed in Table S1 along with additional information such as gene identification, number of amino acids, genomic sequence, molecular weight, isoelectric point of proteins, and localization prediction. The length of AP2/EREBP family genes ranged from 1516 to 10,130 base pairs, and they were located on 10 of the 12 chromosomes in rice. Chromosome 6 had the highest number of genes with 19, while chromosome 12 had the lowest with only two genes. Chromosome 3 has 15 genes, followed by chromosomes 1 and 2 with 14 genes each, and chromosomes 5 and 8 with 13 genes each. Chromosomes 7 and 9 have 10 and five genes, respectively. The PSORT program predicted that most AP2/EREBP proteins were located in the nucleus with a probability greater than 0.5. Some genes, such as *Os05g29810*, *Os08g43200*, and *Os08g43210*, had a probability of up to 0.96 of being located in the nucleus, but there were also proteins localized in the cytoplasm (CS), mitochondria (M), and endoplasmic reticulum (ER). Only the *Os06g11860* protein was located in the extracellular space, including the cell wall (EX). The cDNA length of the genes ranged from 316 to 657, while the protein length ranged from 22 to 917 amino acids. The predicted *pI* value ranged from 4.1 to 11.91, and the predicted molecular mass ranged from 2.42 to 100.08 kDa.

### 2.2. AP2 Family Analysis among Different Species Screening of AP2 Gene Family in Rice, Maize, and Arabidopsis thaliana

**Figure 2:**
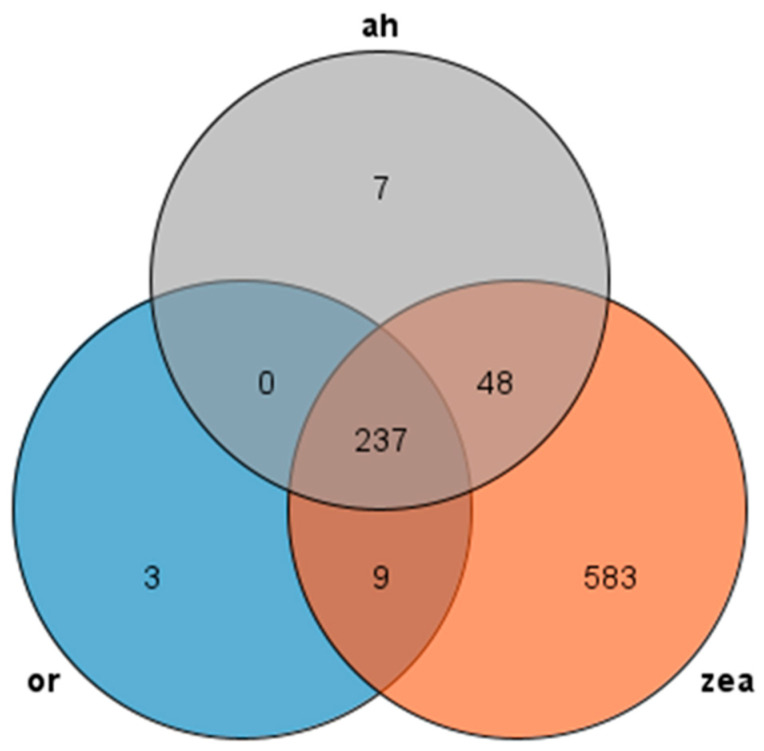
Venn diagram of AP2 gene family: or (rice), zea (*Zea mays*), and ah (*Arabidopsis thaliana*) are all in different colors.

We used Orthofinder to make an orthogroup analysis and identified AP2/EREBP family sequences from the genomes of Rice, *Arabidopsis thaliana*, and *Zea mays* and generated Venn diagrams to compare them (Figure 2). Our analysis revealed 237 transcripts presented in all three species, with most AP2 families in *Arabidopsis thaliana* and rice showing similarity to maize, while rice had only three unique transcripts and maize had 583 unique transcripts. These findings suggest that the AP2 family is widely distributed in Rice, *Zea mays*, and *Arabidopsis thaliana*, and these genes may participate in similar pathways and have similar functions in rice and *Arabidopsis thaliana*.

### 2.3. A Collinearity Analysis of AP2 in Rice

**Figure 3:**
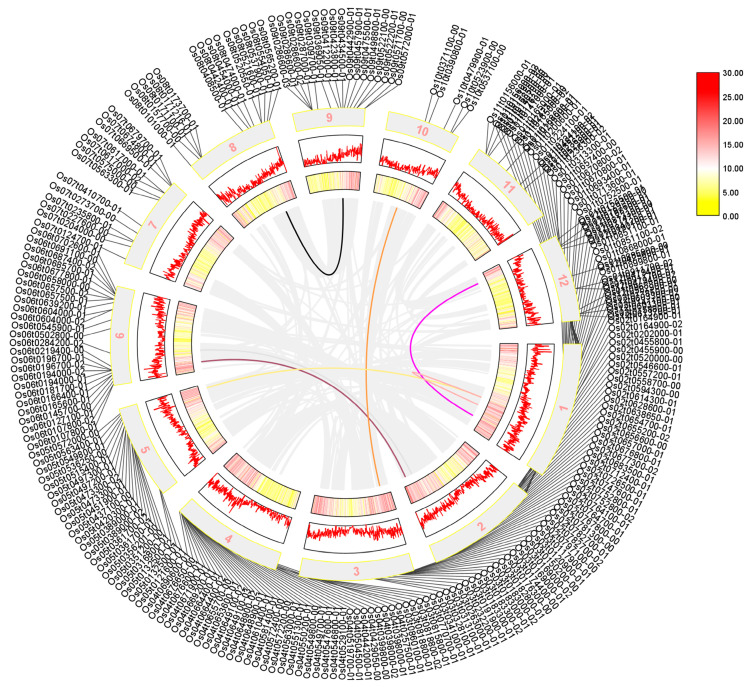
The collinearity analysis of the rice AP2 gene family is presented in a diagram where the outer circle shows the screened transcripts. The mid-term numbers represent the corresponding chromosomes, and rice tandem repeat genes are shown as gray lines in the figure. The tandem genes of the rice AP2 gene family are represented by the colored lines. Legend in Figure 3 represents the relative density of genes on chromosomes.

We performed a collinearity analysis of 273 sequences shared by *Arabidopsis thaliana*, rice, and maize, which were identified using a Venn diagram. Our results in Figure 3 showed that the AP2/EREBP family was widely distributed across all chromosomes of rice, although their positions on the chromosome do not correlate significantly. Additionally, we found that several sequences, including *LOC_Os01g48060*, *LOC_Os01g64790*, *LOC_Os01g48060*, *LOC_Os10g25170*, *LOC_Os02g49460*, *LOC_Os02g55380*, and the sequences *LOC_Os01g54990*, *LOC_Os12g07030*, *LOC_Os05g48870*, *LOC_Os03g08470*, *LOC_Os06g17390*, and *LOC_Os06g08340*, exhibited a high degree of homology, suggesting that was a possible substitution or variation during evolution.

### 2.4. AP2 Gene Family Analysis

**Figure 4:**
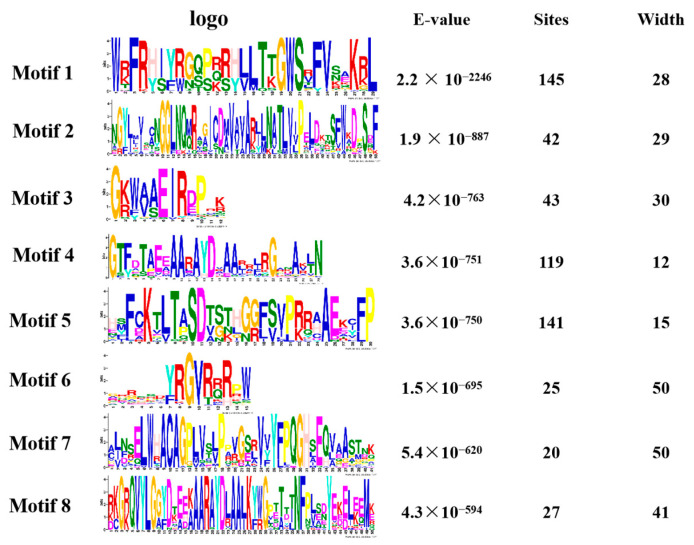
The motif analysis of the AP2 gene family is presented in a picture. The first column represents its conserved sequence, the second column is the E-value < 0.05 and the third and fourth columns show its site and width.

We performed a gene family analysis of the 273 similar AP2/EREBP family members obtained via a Venn diagram. Our analysis revealed that most of the AP2 family members contained eight different conserved sequences, with motif 1, motif 4, and motif 5 appearing multiple times in the sequences (Figure 4). These motifs may be important for the AP2 family to perform functions. Furthermore, our analysis of the conserved domains of the AP2 family identified 17 conserved domains. In Figure S1, we also identified 27 transcripts containing the AP2 domain, four transcripts belonging to the RAV family, and 31 transcripts containing a conserved B3 domain [47].

### 2.5. Comparison of Cold-Regulated AP2/EREBP Family Genes in Chaling Wild Rice and Pei’ai 64S

**Figure 5:**
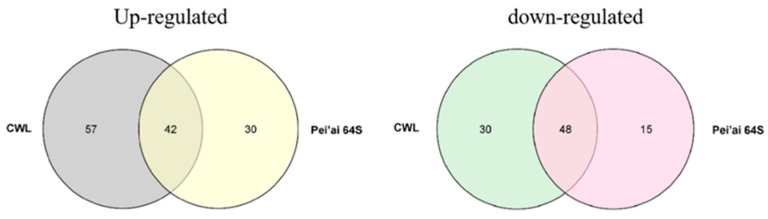
A total of 135 AP2 genes were identified as either cold-induced or cold-repressed in Chaling wild rice and Pei’ai 64S. The AP2 genes from Chaling wild rice are presented in two alignment charts, with the left side representing the up-regulated gene alignment chart and the right side representing the down-regulated gene alignment chart.

**Figure 6:**
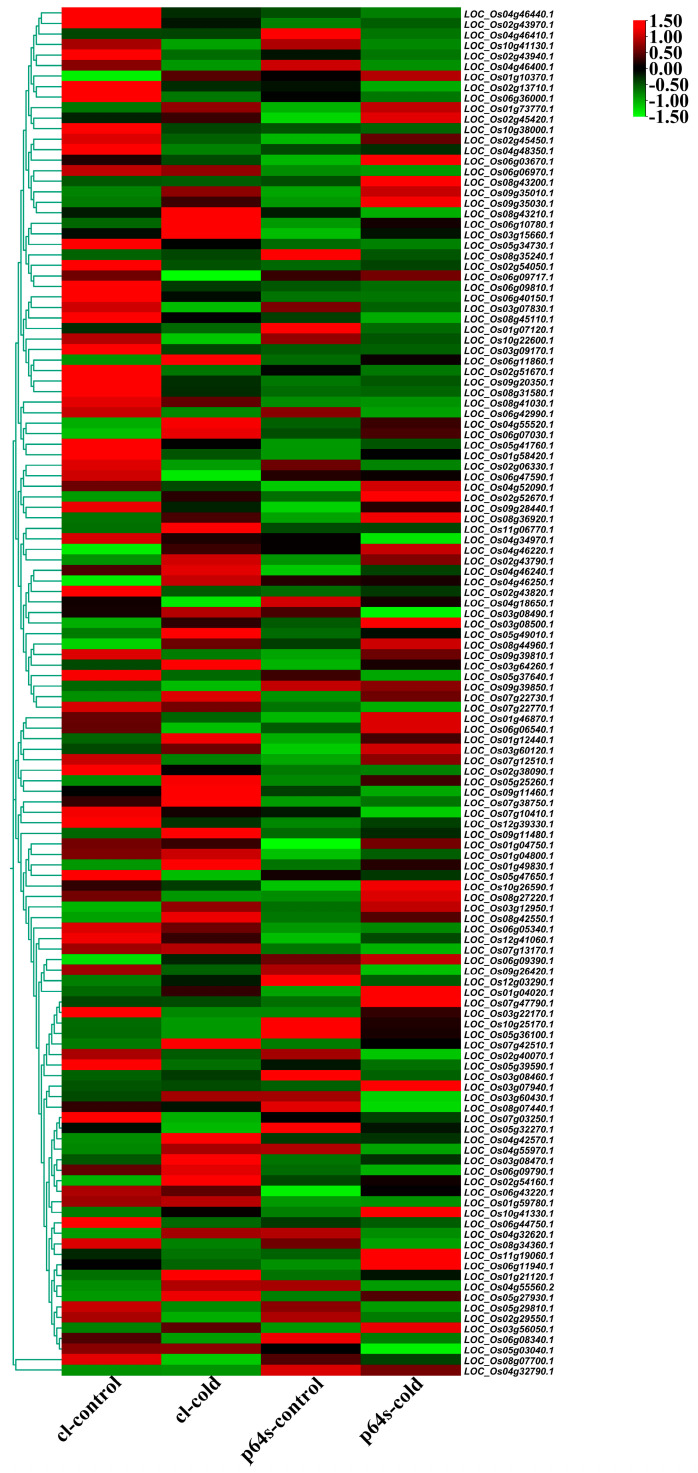
Hierarchical clustering of wild rice and Pei’ai 64S AP2/EREBP family genes expression in cold treatment. The color scale represents a log_2_-fold change.

The expression of AP2/EREBP family genes of Chaling wild rice and Pei’ai 64S were analyzed by using Affymetrix array technology. As shown in Figure 5 and Figure 6, after cold stress, the expression of 42 genes increased in both Chaling wild rice and Pei’ai 64S, 15 genes only increased in Chaling wild rice after cold stress, and 30 genes only increased in Pei’ai 64S. The expression of 48 genes decreased in both Chaling wild rice and Pei’ai 64S, 30 genes decreased in Chaling wild rice, and 15 genes decreased in Pei’ai 64S. These results indicate that AP2 family genes play roles in the regulatory pathway in the cold stress in both wild rice and cultivated rice. Although the AP2 family genes play a regulatory role in the cold stress of wild rice and cultivated rice, their expression levels are different. As shown in Table 1, the total growth rate of 37 genes is higher in Chaling wild rice than that of cultivated rice in cold stress. *Os01g21120* is the largest increase rate gene in Chaling wild rice, which has increased 54 times at low temperatures, but only 18 times in Pei’ai 64S. An asterisk indicates that the increase rate in Chaling wild rice is higher than that in Pei’ai 64S. We analyzed the sequence of the different expression AP2 genes in the transcriptome (Figure S2) and found that it contains three major conserved sequences and that most of the genes in its gene sequence contain AP2 and B3 structural domains. And we analyzed the AP2 gene family and found that most of the genes in the AP2 gene family contain AP2 and B3 conserved structural domains, indicating that the gene sequence in the transcriptome is related to the AP2 gene family.

### 2.6. Sequence Analysis of Promoters of AP2/EREBP Genes

To investigate the possible explanation for abiotic stress-responding genes, promoter sequences about 2 kb upstream of the translational start site were analyzed. The results from both the Plant-CARE and PLACE databases in Table 2 showed that 21 *cis*-regulatory elements (CREs) were identified in rice and responded to abiotic stress. Interestingly, these CREs did not contain common eukaryotic regulatory elements such as TATA and CAAT boxes. For example, the following CREs were found in specific genes: ARE, GA-motif, LTR, Skn-1_motif, and circadian were found in *Os01g49830*, *Os03g08470*, and *Os03g64260*. 5UTR Py-rich stretch, ACE, GT1-motif, HSE, and MRE were found in *Os03g08470* and *Os03g64260*. GCN4_motif, MBS, TGACG-motif, and as-2-box were found in *Os01g49830* and *Os03g64260*, while ABRE, ERE, O2-site, and TC-rich repeats were only found in *Os03g64260*.

**Figure 7:**
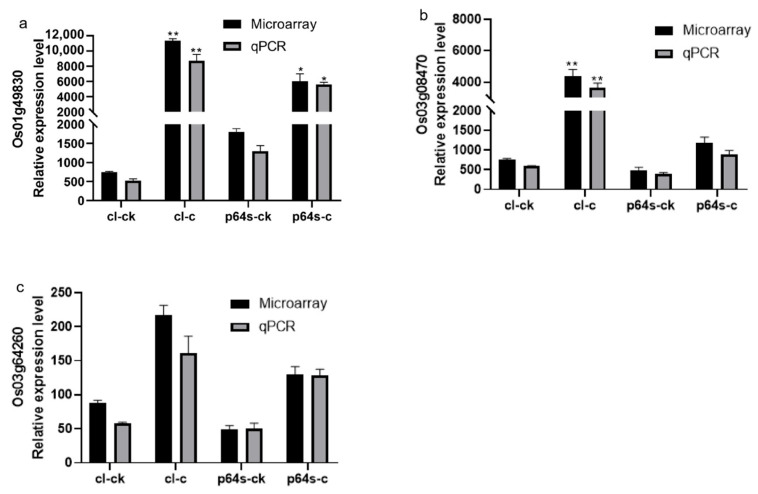
The expression of genes *Os03g08470*, *Os01g49830*, and *Os03g64260* under cold stress is presented in a diagram. The gene expression levels are represented by the ordinates and cl-ck, p64s-ck denotes Chaling wild rice and Pei’ai 64S under normal conditions, whereas cl-c, p64s-c denotes Chaling wild rice and Pei’ai 64S under cold stress. The error bars in the figure represent the mean ± standard error, * represents the difference, *p* < 0.05, and ** represents the significant difference, *p* < 0.01. (**a**) The gene expression of *Os01g49830*. (**b**) The gene expression of *Os03g08470*. (**c**) The gene expression of *Os03g64260*.

The transcription factor ICE1 was shown to bind to the MYC sequence in AP2/EREBP family gene promoters, leading to the regulation of their transcription [48]. In Table 3, we found MYC motif was different in Chaling wild rice and Pei’ai 64S due to a single nucleotide polymorphism (SNP) in three specific genes (*Os01g49830*, *Os03g08470*, *Os03g64260*). It has been suggested that this difference may contribute to weaker AP2/EREBP family gene induction in Indica rice compared to Japonica rice [49]. The expression increased in both Chaling wild rice and Pei’ai 64S under cold stress, with gene expression increasing more in Chaling wild rice than in Pei’ai 64S (Figure 7). This suggested that Chaling wild rice may have a stronger response to cold stress.

### 2.7. Conservation of Upstream and Downstream AP2/EREBP Gene Family

**Figure 8:**
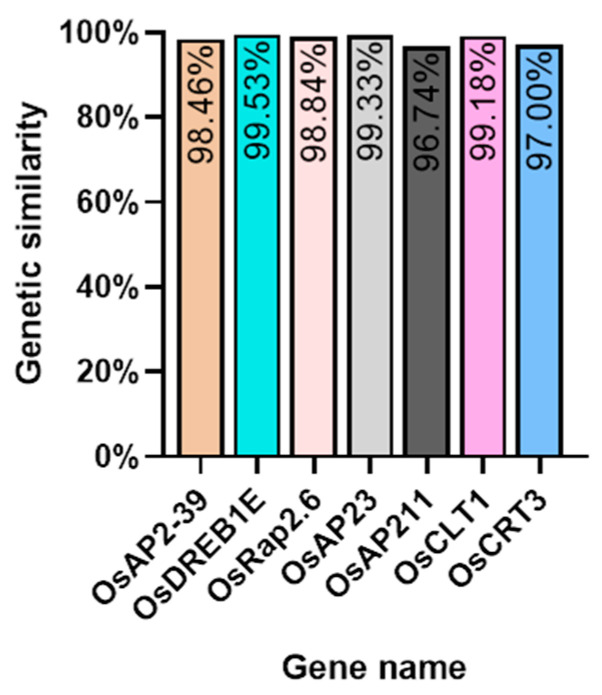
The Nip genome alignment results in the pathway upstream and downstream genes of the AP2/EREBP gene family.

**Figure 9:**
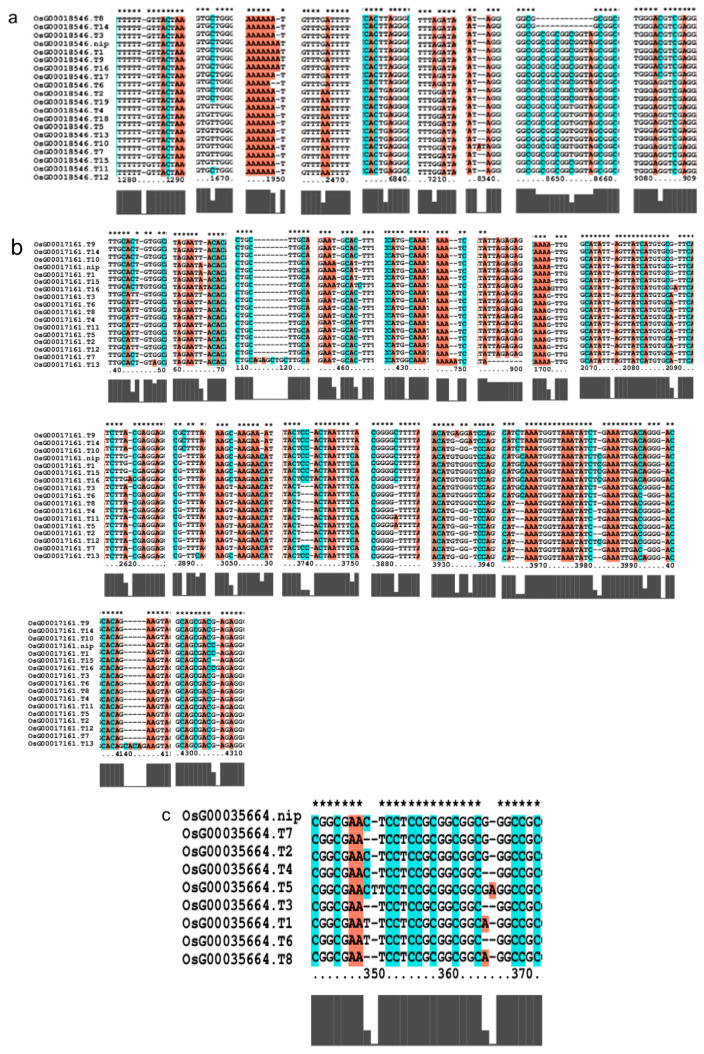

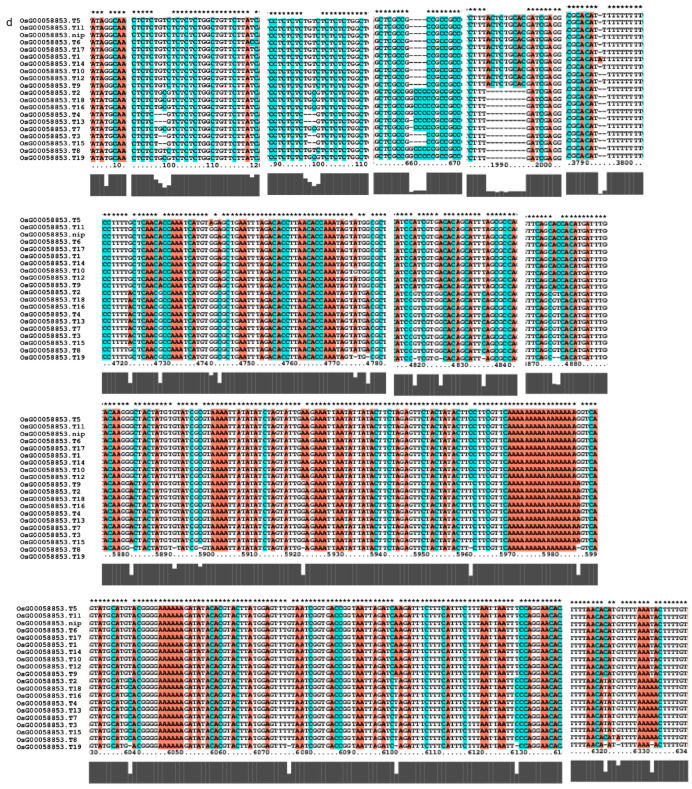

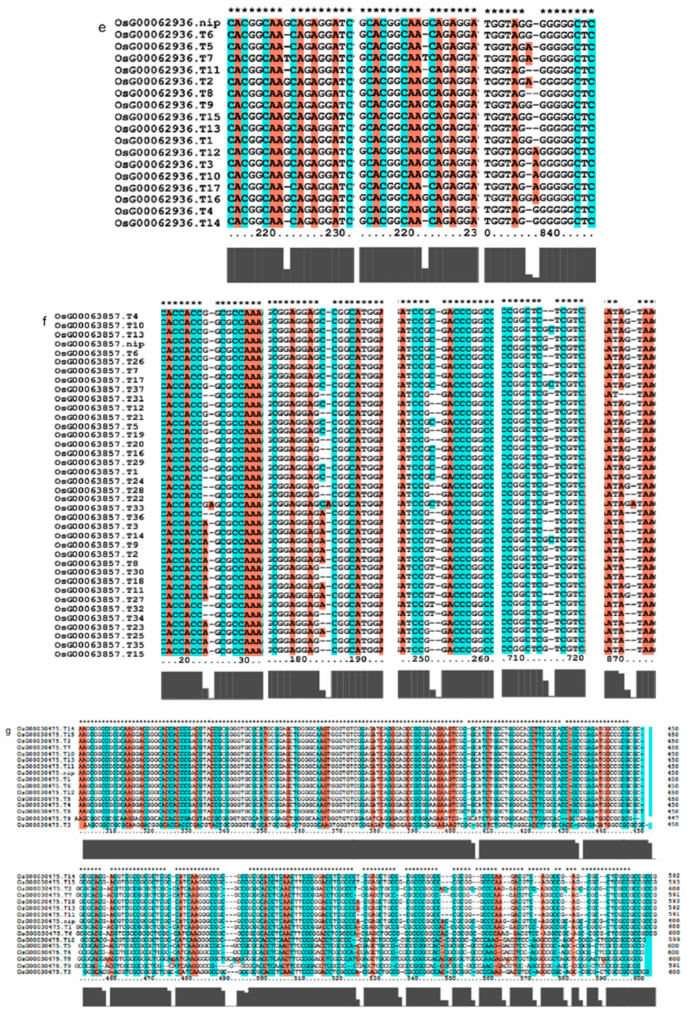
Gene alignment in the regulatory network analysis of the AP2 gene family. * showed all the bases at this site are identical. The genes (**a**) The results of gene OsCLT1 blast. (**b**) The results of gene OsCRT3 blast. (**c**) The results of gene OsAP23 blast. (**d**) The results of gene OsRap2.6 blast. (**e**) The results of gene OsDREB1E blast. (**f**) The results of gene OsAP2-39 blast. (**g**) The results of gene OsAP211 blast.

We investigated several elements of the signaling pathway that respond to the AP2 family in *Oryza sativa*, including *OsAP211*, *OsAP2-39*, *OsDREB2B*, *OsRap2.6*, and other gene families. Our analysis revealed that the overall conservation of these elements was high, with more than 95% similarity across different genotypes (Figure 8). However, we also found that the sequences of upstream transcription factors could vary (Figure 9). Specifically, *OsAP211* and *OsRAP2.6* exhibited the most pronounced changes, with 14 and 28 mutation sites, respectively, while *OsDREB2B* had only two mutation sites. These findings suggest that *OsAP211* and *OsRAP2.6* may be more prone to mutations during rice evolution, whereas *OsDREB2B* and other genes may be more conserved during evolution. Regarding downstream regulatory genes of AP2, the *COR* genes, and *DRE/CRT*, we observed that *OsCRT3* had 24 obvious gene difference sites, while *OsCLT1* was more conservative, with only nine obvious difference sites.

### 2.8. Comparison of Upstream and Downstream Genes of Six Rice Species

**Figure 10:**
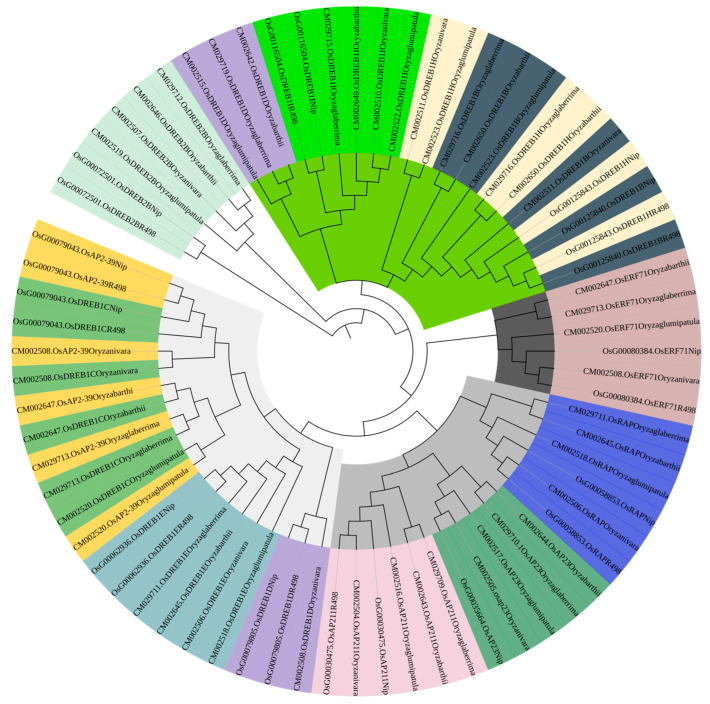
The phylogenetic tree of upstream genes in the AP2 gene family across different rice species is presented in the diagram. The tree was generated using the neighbor-joining method with bootstrap sampling (1000 replicates) and MEGA software version 11. The same color represents the same gene in six rice species.

**Figure 11:**
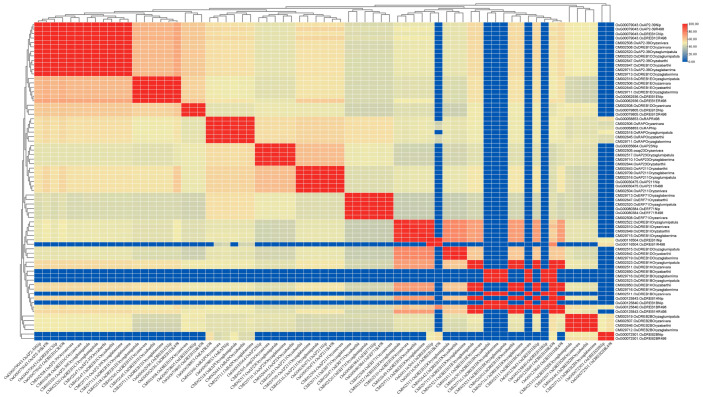
The percentage identity matrix analysis of upstream genes in the AP2/EREBP gene family is presented in the diagram. The gene names are shown on the upper and lower sides of the matrix. The redder the color in the figure, the higher the degree of similarity, while the bluer the color, the less similar.

PleWe downloaded and compared upstream regulatory genes of the AP2 family from six different rice species using the NCBI database, constructed a Phylogenetic tree to study their conservation and evolution (Figure 10), and analyzed the sequence of the AP2 family (Figure 11). Our analysis revealed that the majority of upstream regulatory genes were highly conserved during evolution, and the gene sequences of the same gene in different rice species were more similar and closely related. Our findings provide valuable insights into the conservation and evolution of upstream regulatory genes of the AP2 family across different rice species.

### 2.9. Differential Regulation of AP2 Pathway Genes in Recombinant Inbred Lines from Nipponbare Crossing with Chaling Wild Rice (NC)

**Figure 12:**
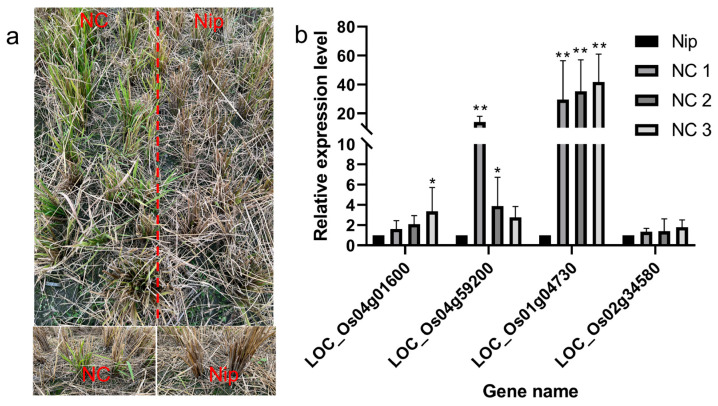
(**a**) Recombinant inbred lines from Nipponbare crossing with Chaling wild rice in winter; (**b**) results of expression level of AP2 signaling pathway genes in recombinant inbred lines from Nipponbare crossing with Chaling wild rice. The error bars in the figure represents the mean ± standard error, * represents the difference, *p* < 0.05, and ** represents the significant difference, *p* < 0.01.

Recombinant inbred lines from Nipponbare crossing with Chaling wild rice can survive cold winters like Chaling wild rice. we analyzed the upstream genes of the AP2 signaling pathway by RT-PCR and found that the expression of the related genes was significantly higher than that of Nip, and the expression of the gene *Os01g04730* was significantly higher than that of Nip among the three lines of NCs, and the expression of the gene *Os04g59200* was significantly higher than the expression of the other groups in NC1 (Figure 12), which indicated that the genes of the AP2 pathway had an important role in the process of cold resistance in rice.

## 3. Discussion

Rice is the main source of food in China, but its growth is often hindered by environmental stress, including low temperatures that can cause reduced yield and spikelet infertility. To adapt to these conditions, rice has developed several regulatory networks [50], such as the AP2/EREBP gene family that plays a vital role in plant stress response by regulating spikelet and floral organ development, flowering time, root development, hormone balance, nutrient efficiency, and biotic and abiotic stress responses [51,52,53,54,55]. Gene family and collinearity analyses revealed that *LOC_Os01g48060* and *LOC_Os01g64790*, *LOC_Os01g48060* and *LOC_Os10g25170*, and *LOC_Os02g49460* and *LOC_Os02g55380* are closely linked, indicating a possible evolutionary relationship within the AP2/EREBP family. Analysis of AP2 gene family promoters and their number, we found AP2/EREBP family genes are induced by cold stress, and their promoter regions are rich in 5UTR Py-rich stretch, ABRE, ARE, ERE, MBS, and TGACG-motif, indicating that these genes are induced by JA and abiotic stress, which is consistent with previous research [56]. The MYC binding site is essential for the binding of ICE1 to the CBF/REB promoter [43,57]. It was found that in the promoters of AP2/EREBP family genes (*Os01g49830*, *Os03g08470*, *Os03g64260*), there were few MYC-binding sites in Chaling wild rice, but these genes were present in Pei’ai64S due to the presence of SNPs, and it was statistically found that the AP2 family was enriched in ABRE as well as TGACG-motif elements, presumably the number of these elements is related to the function in cold resistance. The AP2 family was found to be rich in ABRE and TGACG-motif elements, and it was hypothesized that the number of these elements was related to the function of cold resistance. We utilized Affymetrix array technology to investigate the gene expression changes of the two genotypes under cold stress. The microarray data showed that the expression of AP2/EREBP genes was changed in both Chaling wild rice and Pei’ai64S under cold stress. There were 57 up-regulated genes and 30 down-regulated genes in Chaling wild rice, 30 up-regulated genes, and 15 down-regulated genes in Pei’ai64S, with 42 up-regulated genes and 48 down-regulated genes between them. Gene family analysis revealed that AP2 as well as B3 structural domains are the most prominent conserved structural domains in the AP2 family, and we analyzed the co-up-regulated genes among them and found that all of them contain AP2 or B3 structural domains. Comparing the transcriptomic data and quantitative data of Chaling wild rice and Pei’ai64S, we hypothesized that the up-regulation of some genes in wild rice affects the related cold resistance genes in rice. At the same time, we found that some of our speculated related for AP2-related upstream and downstream genes were up-regulated in cultured wild rice by recombinant self-cross lines, in which the *Os01g04730* gene was up-regulated more than 40-fold, and we still found a large number of highly expressed genes in Chaling wild rice, especially *Os01g21120* had the highest expression in Chaling wild rice, which increased 54-fold under cold stress, while In particular, *Os01g21120* was the most highly expressed gene in Chaling wild rice, with a 54-fold increase in expression under cold stress, whereas in Pei’ai 64S, it only increased 18-fold. This suggests that cold resistance in wild rice is closely related to the genes of the AP2 family. In order to investigate the specific differences between upstream and downstream of the AP2 gene family, we investigated the AP2-related signaling pathway and compared the upstream and downstream genes of the pathway, and found that the regulatory genes were conserved in different rice varieties and that the upstream genes were more conserved than the downstream genes in different rice varieties. The quantitative data showed that the expression of the upstream genes was significantly increased in both wild rice and Nip under cold conditions, indicating that the relevant genes in the upstream regulatory network were also up-regulated. Therefore, we found the conserved structural domains of AP2 as well as B3 possessed by the AP2 gene family via comparative analysis and found that the related AP2 family had a higher expression under cold conditions via quantitative data and transcriptome data analysis, suggesting that the AP2 gene family plays a certain function in cold resistance of wild rice, but in order to make sure whether it is other genes affecting the changes of AP2, we conducted a study on the AP2 genes upstream and downstream genes were compared and found to be more conserved. However, there are still many shortcomings in this paper, exactly how it functions in wild rice still needs further research, as well as the gene *Os01g04730* screened in this paper in overexpression whether it can improve the cold tolerance of rice, still needs further research.

## 4. Materials and Methods

### 4.1. Plant Materials and Conditions

Recombinant inbred lines from Nipponbare crossing with Chaling wild rice (NC) were constructed. Twenty plants capable of over-wintering were selected in each generation up to F10 generation. The seeds of cultivated rice Pei’ai 64S (*Oryza sativa* L.) and Chaling wild rice (provided by Professor Xu Mengliang of Hunan Normal University) were suspended in a sterile solution of 0.1% Hgcl_2_ for 10 min and were washed four times using distilled water and immersed for 3 days under 25 °C, then the seeds were germinated at 37 °C for 3 days in running water. They were partly sown in pots that were put in the net room of the Rice Research Institute of Sichuan Agricultural University. At a five-leaf stage, plants were divided into one control and one treatment group, the control group was maintained under normal growth conditions and the treatment groups were exposed to cold stress (4 °C 12 h). Four countdown second leaves were collected from the treatment and control group. All the biological and technical replicates have been carried out three times.

### 4.2. Extraction of Total RNA and Quantitative PCR

The total RNA was extracted and isolated using TRIzol. Extract method using TRIzol reagent. The procedure was carried out according to the previously described protocols by [58] and The qRT-PCR was performed using an ABI7900 and TBGreen Premix Ex TaqTM II (Tli RNaseH Plus). Each reaction mixture (10 mL) contained 2× Master Mix (5 mL), 0.3 mL of each primer (10 mmol/L), 1 mL of template RNA sample (40 ng), and 3.4 mL of RNase-free water. The thermal cycling program was as follows: initial denaturation for 5 min at 95 °C and 40 amplification cycles (15 s at 95 °C, 40 s at 58 °C, and 20 s at 72 °C). The raw data of RT-PCR were obtained by Bio-Rad CFX Manager, and the relative expression levels were calculated using the 2^−Ct^ method. Ubiquitin 5 (*LOC_Os01g22490*) was used as the internal reference gene in this experiment [59].

### 4.3. Microarray Data Analysis

The procedure was carried out according to protocols previously described by Chen et al. [58]. The IDs of probe sets presented on the Affymetrix rice genome array representing the AP2/EREBP family genes were identified using the Rice Multi-platform Microarray Search tool [60]. Hierarchical clustering was generated with Cluster using normalized log ratios [61].

### 4.4. Analysis of AP2 Protein in Rice

All the AP2 family genes proteins were searching by GRAMENE (http://www.gramene.org/ (accessed on 12 April 2020)), the molecular weights and isoelectric points of AP2 were predicted by the Compute pI/Mw tool program (http://web.expasy.org/compute_pi/ (accessed on 12 April 2020)), and the program PSORT was used for the localization prediction (http://psort.ims.u-tokyo.ac.jp (accessed on 12 April 2020)).

### 4.5. Promoter Analysis

DNA sequences of rice AP2/EREBP genes and 1500 bp ahead of the translation initiation codon (ATG) were collected from the GRAMENE database. The AP2/EREBP family genes promoter was analyzed by the Plant-CARE database (PlantCARE, a database of plant promoters and their *cis*-acting regulatory elements (ugent.be (accessed on 14 June 2023)), and uses the PLACE database to verify the results.

### 4.6. AP2 Family Analysis among Different Species

We used TBtool to extract the transcripts of *Arabidopsis thaliana*, *Oryza sativa*, and *Zea mays* from the previously screened AP2/EREBP gene family genes. (FTP Download (ensembl.org (accessed on 27 March 2023))) and used OrthoFinder software (version 2.2.7) to infer direct homology in rice maize as well as in *Arabidopsis* [62]. Next, we identified the common transcripts and all candidate AP2 protein sequences were submitted to NCBI-CDD (Welcome to NCBI Batch CD-search (nih.gov (accessed on 27 March 2023)), and MEME (MEME-Submission form (meme-suite.org (accessed on 27 March 2023)) [63] to obtain their conserved structural domains and conserved characteristics, and the conserved structural domains, conserved features, and gene structures were mapped using TBtool [64]. with E-value < 1 × 10^−5^, Covariance analysis was performed using TBtool’s one step MCscanX and plotted with Advanced Circos.

### 4.7. Analysis of Upstream Gene of AP2 Family

We retrieved the upstream and downstream genes of the AP2/EREBP gene family from the Molecular Breeding Knowledge Base database (MBKbase, Genotype) and analyzed their variations across different genotypes. Additionally, we obtained the upstream and downstream gene sequences of the AP2/EREBP gene family from various rice varieties in the NCBI database (*Oryza sativa* (ID 10)-Genome-NCBI (nih.gov (accessed on 10 February 2023)), sequence comparison using ClustalX 2.1 and construction of evolutionary trees using MEGA (version 11).

## 5. Conclusions

In this study, we analyzed selected AP2 gene families as well as their promoters to identify conserved sequences, structural domains, and response elements. We also found that the AP2 gene family is highly expressed in wild rice by microarray data and quantitative analysis under cold conditions. We compared the upstream and downstream pathway differences of AP2 genes and found that the upstream genes were more conserved, and quantitatively found that their upstream genes were higher in wild rice under cold conditions. Some of the AP2 family gene is highly expressed in recombinant inbred lines from Nipponbare crossing with Chaling wild rice.

## Figures and Tables

**Table 1:** List of differentially expressed AP2 genes wild-rice and Pei’ai 64S under cold stress and control (CT). The values in the table represent significant fold change values ≥ 2-fold (with corrected *p*-value ≤ 0.05). An asterisk indicates that the expression pattern in Chaling wild rice is higher than that in Pei’ai 64S.

	Gene Identifier	Gene Description	[WT CD] vs. [WT CT]		[PEI’AI 64S DS] vs. [PEI’AI 64S CT]	
			Fold Change	Regulation	Fold Change	Regulation
	LOC_Os01g04020	Putative uncharacterized protein	2.08	up	5.42	up
	LOC_Os01g04750	Putative AP2/ERF and B3 domain-containing protein	0.87	down	15.01	up
	LOC_Os01g04800	AP2/ERF and B3 domain-containing protein	1.17	up	2.31	up
	LOC_Os01g07120	Dehydration-responsive element-binding protein 2A	0.89	down	0.56	down
*	LOC_Os01g10370	Ap21	3.88	up	1.4	up
	LOC_Os01g12440	Os01g0224100 protein	4.52	up	17	up
*	LOC_Os01g21120	Os01g0313300 protein	54.57	up	18.67	up
	LOC_Os01g46870	Ap24	0.52	down	4.01	up
*	LOC_Os01g49830	AP2/ERF and B3 domain-containing protein	15.5	up	3.5	up
	LOC_Os01g54890	Os01g0752500 protein	0.19	down	0.06	down
	LOC_Os01g58420	Ethylene-responsive element binding factor3	0.42	down	1.82	up
	LOC_Os01g59780	Os01g0813300 protein	1.07	up	1.35	up
	LOC_Os01g67410	Os01g0899800 protein	0.58	down	0.56	down
	LOC_Os01g73770	Dehydration-responsive element-binding protein 1F	4.5	up	27.73	down
	LOC_Os02g06330	unknow	0.15	down	0.27	down
	LOC_Os02g13710	Os02g0231000 protein	0.34	down	0.12	down
	LOC_Os02g29550	Os02g0499000 protein	0.43	down	0.51	down
	LOC_Os02g38090	Enhancer of shoot regeneration ESR1-like protein	0.47	down	0.91	down
	LOC_Os02g40070	Os02g0654700 protein	0.35	down	0.05	down
*	LOC_Os02g43790	Os02g0654700 protein	22.15	up	21.93	up
	LOC_Os02g43820	Os02g0655200 protein	0.5	down	1.14	up
	LOC_Os02g43940	Os02g0656600 protein	0.1	down	0.26	down
	LOC_Os02g43970	Os02g0657000 protein	0.46	down	1.26	up
	LOC_Os02g45420	Os02g0676800 protein	1.08	up	1.42	up
	LOC_Os02g45450	Dehydration-responsive element-binding protein 1G	0.25	down	33.88	up
	LOC_Os02g51670	Os02g0752800 protein	0.23	down	0.51	down
*	LOC_Os02g52670	AP2 domain-containing transcription factor-like	6.78	up	2.23	up
	LOC_Os02g54050	Os02g0781300 protein	0.6	down	1.07	up
	LOC_Os02g54160	Ethylene-responsive transcription factor 1	2.37	up	1.24	up
	LOC_Os03g05590	AP2 domain-containing protein, expressed	0.42	down	0.52	down
	LOC_Os03g07830	Dehydration-responsive element-binding protein 2E	0.22	down	0.49	down
	LOC_Os03g07940	Putative uncharacterized protein	1.17	up	9.92	up
*	LOC_Os03g08460	AP2 domain-containing protein, expressed	1.23	up	0.31	down
*	LOC_Os03g08470	AP2 domain-containing protein	5.9	up	2.44	up
*	LOC_Os03g08490	Os03g0183200 protein	1.12	up	0.74	down
	LOC_Os03g08500	AP2 domain-containing protein, expressed	1.82	up	1.89	up
	LOC_Os03g09170	AP2 domain-containing protein, expressed	0.17	down	0.87	down
*	LOC_Os03g12950	ANT, putative, expressed	6.78	up	3.27	up
	LOC_Os03g15660	AP2 domain-containing protein, expressed	1.54	up	1.59	up
	LOC_Os03g22170	AP2 domain-containing protein, expressed	0.28	down	2.31	up
	LOC_Os03g56050	Os03g0770700 protein	7.06	up	38.47	up
	LOC_Os03g60120	AP2 domain-containing protein, expressed	1.53	up	2.97	up
*	LOC_Os03g60430	Os03g0818800 protein	2.26	up	0.099	up
	LOC_Os03g64260	AP2 domain-containing protein, expressed	2.41	up	2.93	up
	LOC_Os04g18650	B1234D02.6 protein	0.25	down	0.69	down
*	LOC_Os04g32620	OSJNBa0039C07.10 protein	1.51	up	0.67	down
	LOC_Os04g32790	OSJNBb0014D23.10 protein	0.83	down	0.75	down
	LOC_Os04g34970	OSJNBa0042L16.9 protein	0.73	down	0.41	down
*	LOC_Os04g42570	Os04g0504500 protein	3.37	up	1.03	up
*	LOC_Os04g46220	Development-related ERF protein	1.73	up	1.25	up
	LOC_Os04g46240	Putative uncharacterized protein	1.28	up	1.71	up
*	LOC_Os04g46250	Ap26	2.28	up	0.99	up
*	LOC_Os06g46410	Auxin response factor 17	1.02	up	0.12	down
	LOC_Os04g46400	OSJNBb0034G17.6 protein	0.32	down	0.49	down
	LOC_Os04g48350	Dehydration-responsive element-binding protein 1E	0.02	down	1.37	up
	LOC_Os04g52090	OSJNBa0085I10.10 protein	0.57	down	5.5	up
*	LOC_Os04g55520	OSJNBa0010D21.10 protein	2.6	up	1.48	up
*	LOC_Os04g55560	Os04g0649100 protein	3.25	up	0.28	down
*	LOC_Os04g55970	Os04g0653600 protein	3.6	up	0.2	down
*	LOC_Os05g03040	Os05g0121600 protein	1	up	0.42	down
*	LOC_Os05g25260	Os05g0316800 protein	9.98	up	5.33	up
*	LOC_Os05g27930	unknown	4.85	up	2.68	up
	LOC_Os05g29810	Os05g0361700 protein	0.08	down	0.03	down
	LOC_Os05g32270	Os05g0389000 protein	0.58	down	0.6	down
	LOC_Os05g34730	Os05g0420300 protein	0.34	down	0.36	down
	LOC_Os05g36100	Os05g0437050 protein	0.71	down	0.6	down
	LOC_Os05g37640	Os05g0448675 protein	0.19	down	0.08	down
	LOC_Os05g39590	Dehydration-responsive element-binding protein 2D	0.13	down	0.36	down
	LOC_Os05g41760	Os05g0497200 protein	0.48	down	1.86	up
	LOC_Os05g41780	Os05g0497300 protein	0.49	down	3.43	up
	LOC_Os05g47650	Os05g0549800 protein	0.31	down	0.8	down
*	LOC_Os05g49010	Os05g0564700 protein	5.51	up	1.89	up
	LOC_Os06g03670	Dehydration-responsive element-binding protein 1C	0.53	down	25.16	up
	LOC_Os06g05340	Os06g0145700 protein	0.81	down	1.08	up
	LOC_Os06g06540	Putative uncharacterized protein	0.48	down	1.78	up
	LOC_Os06g06970	Dehydration-responsive element-binding protein 1D	0.87	down	0.58	down
*	LOC_Os06g07030	Os06g0166400 protein	11.59	up	1.94	up
	LOC_Os06g08340	Os06g0181700 protein	0.68	down	0.6	down
*	LOC_Os06g09390	EREBP transcription factor	1.53	up	1.11	up
	LOC_Os06g09717	Putative uncharacterized protein	0.31	down	1.11	up
*	LOC_Os06g09790	Putative uncharacterized protein	1.24	up	0.73	down
	LOC_Os06g09810	Putative uncharacterized protein	0.49	down	0.93	down
	LOC_Os06g10780	Putative uncharacterized protein	4.14	up	4.25	up
*	LOC_Os06g11860	Os06g0222400 protein	1.5	up	1.15	up
	LOC_Os06g11940	Putative uncharacterized protein	0.44	down	10.7	up
	LOC_Os06g36000	Os06g0553700 protein	0.09	down	0.22	down
	LOC_Os06g40150	AP domain containing transcription factor	0.32	down	0.94	down
	LOC_Os06g42990	Putative uncharacterized protein	0.4	down	0.4	down
	LOC_Os06g43220	Os06g0639200 protein	0.86	down	4.53	up
	LOC_Os06g44750	Aintegumenta-like protein	0.19	down	0.74	down
	LOC_Os06g47590	Os06g0691100 protein	0.69	down	0.98	down
	LOC_Os07g03250	Os07g0124700 protein	0.25	down	0.77	down
	LOC_Os07g10410	Os07g0204000 protein	0.63	down	0.34	down
	LOC_Os07g12510	Os07g0227600 protein	0.72	down	1.39	up
*	LOC_Os07g13170	Os07g0235800 protein	1.03	up	0.88	down
*	LOC_Os07g22730	Os07g0235800 protein	2.05	up	1.77	up
	LOC_Os07g22770	Os07g0410700 protein	0.77	down	0.19	down
	LOC_Os07g38750	Os07g0575000 protein	1.75	up	2.46	up
*	LOC_Os07g42510	EREB-like protein	21.1	up	1.077	up
	LOC_Os07g47330	Frizzy panicle	0.55	down	0.4	down
	LOC_Os07g47790	Os07g0674800 protein	0.97	down	11.24	up
	LOC_Os08g07440	Os08g0171100 protein	0.91	down	0.57	down
	LOC_Os08g07700	Os08g0173700 protein	0.32	down	0.7	down
	LOC_Os08g27220	Os08g0360800 protein	0.35	down	3.26	up
	LOC_Os08g31580	AP domain DRE binding factor	0.5	down	1.02	up
	LOC_Os08g34360	AP2-EREBP transcription factor	0.12	down	0.04	down
*	LOC_Os08g35240	AP2 domain transcription factor-like	1.89	up	0.11	down
	LOC_Os08g36920	Os08g0474000 protein	4.62	up	374.69	up
	LOC_Os08g41030	Os08g0521600 protein	0.75	down	0.95	down
*	LOC_Os08g42550	AP2 domain-containing protein AP29-like	4.38	up	2.29	up
	LOC_Os08g43200	Os08g0545500 protein	0.85	down	10.47	up
*	LOC_Os08g43210	Os08g0545500 protein	1.92	up	0.51	down
	LOC_Os08g44960	Putative uncharacterized protein	1.47	up	1.3	up
	LOC_Os08g45110	Dehydration-responsive element-binding protein 2C	0.54	down	0.49	down
*	LOC_Os09g11460	AP2/ERF domain protein	1.49	up	0.77	down
*	LOC_Os09g11480	AP2/ERF domain protein	48.6	up	13.84	up
	LOC_Os09g20350	Os09g0369000 protein	0.36	down	1.29	up
	LOC_Os09g26420	Os09g0434500 protein	0.81	down	0.73	down
	LOC_Os09g28440	Ethylene-binding protein-like	0.42	down	38.84	up
	LOC_Os12g41046	unknown	8.18	up	127.36	up
	LOC_Os12g41047	unknown	6.32	up	119.7	up
	LOC_Os12g41048	unknown	0.2	down	7.3	up
	LOC_Os12g41049	unknown	0.57	down	0.89	down
	LOC_Os12g41050	Putative uncharacterized protein	0.07	down	0.39	down
	LOC_Os12g41051	unknown	0.68	down	0.58	down
	LOC_Os12g41052	unknown	0.75	down	3.14	up
	LOC_Os12g41053	unknown	0.23	down	0.81	down
	LOC_Os12g41054	unknown	0.25	down	0.32	down
	LOC_Os12g41055	unknown	34.3	up	95.17	up
*	LOC_Os12g41056	unknown	4.41	up	1.02	up
	LOC_Os12g41057	unknown	0.29	down	8.66	up
*	LOC_Os12g41058	unknown	2.13	up	0.25	down
	LOC_Os12g41059	unknown	0.6	down	1.18	up
	LOC_Os12g41060	AP2 domain-containing protein, expressed	0.78	down	1.34	up

**Table 2:** The *cis*-element sequence is putative to be located in the 5′ upstream region of AP2/EREBP genes in rice.

*cis*-Regulatory Elements	Function	Numbers
ABRE	*cis*-acting element involved in the abscisic acid responsiveness	587
ACE	*cis*-acting element involved in light responsiveness	39
ARE	*cis*-acting regulatory element essential for the anaerobic induction	261
ERE	ethylene-responsive element	108
GA-motif	part of a light-responsive element	25
GCN4_motif	*cis*-regulatory element involved in endosperm expression	22
GT1-motif	light-responsive element	127
LTR	*cis*-acting element involved in low-temperature responsiveness	77
MBS	MYB binding site involved in drought-inducibility	98
MRE	MYB binding site involved in light responsiveness	47
O2-site	*cis*-acting regulatory element involved in zein metabolism regulation	76
TC-rich repeats	*cis*-acting element involved in defense and stress responsiveness	42
TCA-element	*cis*-acting element involved in salicylic acid responsiveness	66
TGACG-motif	*cis*-acting regulatory element involved in the MeJA-responsiveness	309
circadian	*cis*-acting regulatory element involved in circadian control	30

**Table 3:** *Cis*-element analysis of promoter sequences of AP2 genes in wild rice and Pei’64s.

Genes	Variety	Location	Promoter Sequence
*Os01g49830*	Chaling wild rice	−1038	TCCCTCATCTCCCCTG
	Pei’64s	−1038	TCCCTCATCTGCCCTG
*Os03g08470*	Chaling wild rice	−1598	AATAACGTGTGATTAA
	Pei’64s	−1598	AATAACATGTGATTAA
*Os03g64260*	Chaling wild rice	−961	TGAATCAAAGGAAAAA
	Pei’64s	−961	TGAATCAAATGAAAAA

## Data Availability

Not applicable.

## Supplementary Materials

The following supporting information can be downloaded at https://www.mdpi.com/article/10.3390/ijms241914441/s1.
