# Supplementary material for: AP2/EREBP Pathway Plays an Important Role in Chaling Wild Rice Tolerance to Cold Stress
Source: Int J Mol Sci. 2023 Sep 22;24(19):14441. doi: 10.3390/ijms241914441 (PMC10572191; doi:10.3390/ijms241914441)
Supplement: Supplementary file 1 [file ijms-24-14441-s001.zip › ijms-2578583-supplementary.pdf]

Supplemental Table S1. Prediction of Rice AP2/EREBP Family features and AP2 Protein localization.

Gene name	Gene name(GRAMENE)	Gene lengh	cDNA length	Protein length	Mol.wt.(kDA)	PI	Localization prediction
1	LOC_Os01g04020	3329	1372	309	33.99	4.43	C(0.348);CS(0.261)
2	LOC_Os01g04750	2154	954	317	34.87	7.07	CS(0.314),C(0.304)
3	LOC_Os01g04800	2710	1510	365	40.15	9.82	ER(0.222)
4	LOC_Os01g07120	6022	3638	274	30.14	5.77	N(0.826)
5	LOC_Os01g10370	2096	896	228	25.08	5.98	N(0.696)
6	LOC_Os01g12440	3016	1816	380	41.8	4.83	CS(0.522)
7	LOC_Os01g21120	2190	908	207	22.77	5.06	CS(0.304)
8	LOC_Os01g46870	2297	1076	292	32.12	5.01	ER(0.444);(0.444)
9	LOC_Os01g49830	2733	1533	393	43.23	9.09	CS(0.565)
10	LOC_Os01g54890	2124	924	212	23.32	5.07	N(0.435)
11	LOC_Os01g58420	2261	1061	235	25.85	9.55	CS(0.478)
12	LOC_Os01g59780	2645	384	127	13.97	10.21	CS(0.435)
13	LOC_Os01g67410	5543	2755	695	76.45	6.56	CS(0.348)
14	LOC_Os01g73770	2036	836	219	45.09	5.8	N(0.87)
15	LOC_Os02g13710	1516	316	22	2.42	5.05	CS(0.435)
16	LOC_Os02g29550	8295	2697	222	24.42	8.99	N(0.652)
17	LOC_Os02g38090	2238	1038	345	37.95	7.07	N(0.696)
18	LOC_Os02g40070	3294	1288	321	35.31	7.3	M(0.783)
19	LOC_Os02g43790	2499	1299	303	33.33	6.02	N(0.696)
20	LOC_Os02g43820	2673	774	148	162.8	6.33	M(0.652)
21	LOC_Os02g43940	2601	1401	287	31.57	4.97	N(0.957)
22	LOC_Os02g43970	2238	1038	225	24.75	5.22	N(0.913)
23	LOC_Os02g45420	2891	1691	261	28.71	5.48	N(0.696)
24	LOC_Os02g45450	2472	1272	224	24.64	5.14	N(0.739)
25	LOC_Os02g51670	2854	1654	338	37.18	7.77	N(0.739)
26	LOC_Os02g52670	2111	991	242	26.62	6.53	CS(0.435)
27	LOC_Os02g54050	1983	783	211	23.21	6.19	N(0.696)
28	LOC_Os02g54160	4064	1686	365	40.15	4.89	N(0.87)
29	LOC_Os03g05590	1833	633	141	15.51	6.3	N(0.826)
30	LOC_Os03g07830	2165	965	318	34.98	5.91	N(0.348)
31	LOC_Os03g07940	3530	513	170	18.7	9.82	M(0.522)
32	LOC_Os03g08460	3083	1321	326	35.86	6.15	N(0.957)
33	LOC_Os03g08470	2996	1574	334	36.74	5.84	M(0.565)
34	LOC_Os03g08490	2381	749	177	19.47	4.1	CS(0.394)
35	LOC_Os03g08500	2395	1192	329	36.19	4.56	CS(0.478)

36	LOC_Os03g09170	2523	1323	297	32.67	6.06	N(0.739)
37	LOC_Os03g12950	5503	2510	642	70.62	6.29	N(0.739)
38	LOC_Os03g15660	2335	1135	258	28.38	5.39	N(0.348)
39	LOC_Os03g22170	2455	1164	243	26.73	6.17	N(0.261);CS(0.217)
40	LOC_Os03g56050	4883	2373	339	37.29	6.67	CS(0.522)
41	LOC_Os03g60120	2291	1070	241	26.51	10.21	N(0.348)
42	LOC_Os03g60430	5342	2123	430	47.3	7.75	N(0.652)
43	LOC_Os03g64260	2117	917	255	28.05	6.06	N(0.826)
44	LOC_Os04g18650	2078	878	128	14.08	5.88	N(0.696)
45	LOC_Os04g32620	5560	3762	268	29.48	9.81	N(0.696)
46	LOC_Os04g32790	2229	1029	292	32.12	4.53	N(0.739)
47	LOC_Os04g34970	1935	735	244	26.84	6.92	N(0.522)
48	LOC_Os04g42570	5464	2367	658	72.38	6.05	CS(0.609)
49	LOC_Os04g46220	2732	1532	318	34.98	5.01	C(0.522)
50	LOC_Os04g46240	2157	957	318	34.98	5.11	N(0.826)
51	LOC_Os04g46250	2599	1399	328	36.08	5.33	N(0.739)
52	LOC_Os04g46410	7239	3731	917	100.87	5.85	N(0.652)
53	LOC_Os04g46400	2336	1136	284	312.47	5.23	N(0.870)
54	LOC_Os04g48350	1860	660	219	24.09	5.44	N(0.609)
55	LOC_Os04g52090	2191	991	222	24.42	9.62	CS(0.522)
56	LOC_Os04g55520	2415	1215	233	25.63	9.59	N(0.435)
57	LOC_Os04g55560	4950	2648	93	10.23	6.09	M(0.391)
58	LOC_Os04g55970	5751	2152	495	24.45	5.72	N(0.739)
59	LOC_Os05g03040	4604	1539	512	56.32	6.7	N(0.739)
60	LOC_Os05g25260	2406	1206	281	30.91	4.84	ER(0.556)
61	LOC_Os05g27930	4512	1615	93	10.23	11.1	N(0.739)
62	LOC_Os05g29810	2448	1049	198	21.78	7.07	N(0.913)
63	LOC_Os05g32270	5296	1237	300	33	5.64	N(0.696)
64	LOC_Os05g34730	1794	594	197	21.67	9.67	M(0.394);CS(0.348)
65	LOC_Os05g36100	1989	384	127	13.97	4.37	CS(0.478)
66	LOC_Os05g37640	10130	657	218	23.98	5.56	N(0.565)
67	LOC_Os05g39590	3549	1278	261	28.7	5.06	CS(0.478)
68	LOC_Os05g41760	2115	915	189	20.79	9.81	CS(0.565)
69	LOC_Os05g41780	2293	1093	236	25.96	9.1	ER(0.222);(0.222)
70	LOC_Os05g47650	2378	1178	343	37.73	9.77	M(0.391)
71	LOC_Os05g49010	2049	474	157	17.27	4.05	N(0.444)
72	LOC_Os06g03670	1964	764	214	23.54	5.01	CS(0.478)
73	LOC_Os06g05340	3503	969	322	35.42	5.63	N(0.391)

74	LOC_Os06g06540	2229	1209	342	37.62	4.85	N(0.478)
75	LOC_Os06g06970	2104	904	253	27.83	10.18	N(0.261);CS(0.261)
76	LOC_Os06g07030	2205	1005	184	20.24	10.63	N(0.826)
77	LOC_Os06g08340	2180	873	178	19.58	5.7	N(0.478);M(0.478)
78	LOC_Os06g09390	3782	1676	362	39.82	4.78	N(0.609)
79	LOC_Os06g09717	1824	624	207	22.77	4.79	N(0.652)
80	LOC_Os06g09790	1776	576	191	21.01	4.84	CS(0.435)
81	LOC_Os06g09810	1824	624	207	22.77	4.99	CS(0.565)
82	LOC_Os06g10780	2744	813	270	29.7	5.16	N(0.609)
83	LOC_Os06g11860	2535	692	95	10.45	4.72	EX(0.556)
84	LOC_Os06g11940	4903	1428	475	52.25	5.46	N(0.696)
85	LOC_Os06g36000	1912	712	113	12.43	3.67	CS(0.478)
86	LOC_Os06g40150	2763	1328	243	26.73	9.47	N(0.826)
87	LOC_Os06g42990	2460	1260	419	46.09	10.36	CS(0.435)
88	LOC_Os06g43220	5027	2014	334	36.74	6.46	N(0.783)
89	LOC_Os06g44750	5055	1744	469	51.59	6.43	N(0.783)
90	LOC_Os06g47590	2179	979	188	20.68	6.15	M(0.565)
91	LOC_Os07g03250	5902	1434	331	36.41	7.4	N(0.783)
92	LOC_Os07g10410	2132	932	266	29.26	8.59	N(0.522)
93	LOC_Os07g12510	2049	849	282	31.02	10.12	N(1)
94	LOC_Os07g13170	5568	2000	459	50.49	8.2	N(0.662)
95	LOC_Os07g22730	2158	899	199	21.89	11.24	N(0.739)
96	LOC_Os07g22770	2692	1492	239	26.29	9.33	N(0.826)
97	LOC_Os07g38750	2488	1234	408	44.88	8.87	M(0.394);ER(0.348)
98	LOC_Os07g42510	3052	1433	342	37.62	4.64	CS(0.522)
99	LOC_Os07g47330	2688	1488	318	34.98	6.51	N(0.696)
100	LOC_Os07g47790	2173	879	213	23.43	6.32	CS(0.348
101	LOC_Os08g07440	1600	400	59	6.49	4.78	CS(0.478)
102	LOC_Os08g07700	1995	795	175	19.25	5.4	N(0.826)
103	LOC_Os08g27220	5521	4321	189	20.79	10.66	M(0.478)
104	LOC_Os08g31580	2851	1651	280	30.8	7.03	N(0.739)
105	LOC_Os08g34360	5596	1866	419	46.09	5.41	N(0.739)
106	LOC_Os08g35240	1947	747	243	26.73	4.77	N(0.826)
107	LOC_Os08g36920	2281	834	219	24.09	9.92	N(0.784)
108	LOC_Os08g41030	2100	900	189	20.79	9.18	M(0.565)
109	LOC_Os08g42550	8884	2717	453	49.83	12	N(0.348)
110	LOC_Os08g43200	5998	981	326	35.86	11.91	N(0.913)
111	LOC_Os08g43210	5998	981	326	35.86	11.91	N(0.913)

112	LOC_Os08g44960	1611	411	136	14.96	7.88	N(0.826)
113	LOC_Os08g45110	2351	1151	230	25.3	5.57	N(0.729)
114	LOC_Os09g11460	3099	1515	232	25.52	6.66	N(0.478)
115	LOC_Os09g11480	2758	1107	250	27.5	4.8	N(0.913)
116	LOC_Os09g20350	1695	495	162	17.82	9.99	N(0.522)
117	LOC_Os09g26420	4413	1601	396	43.56	4.88	CS(0.348)
118	LOC_Os09g28440	2300	1100	274	30.14	6.01	CS(0.341)
119	LOC_Os12g41050	3166	327	108	11.88	8.69	CS(0.609)
120	LOC_Os12g41060	5398	2109	323	35.53	4.65	CS(0.522)

The program PSORT was used for the localization prediction (http://psort.ims.u-tokyo.ac.jp). Abbreviations: bp, base pair; aa, amino acids; ID, identification; No., number.

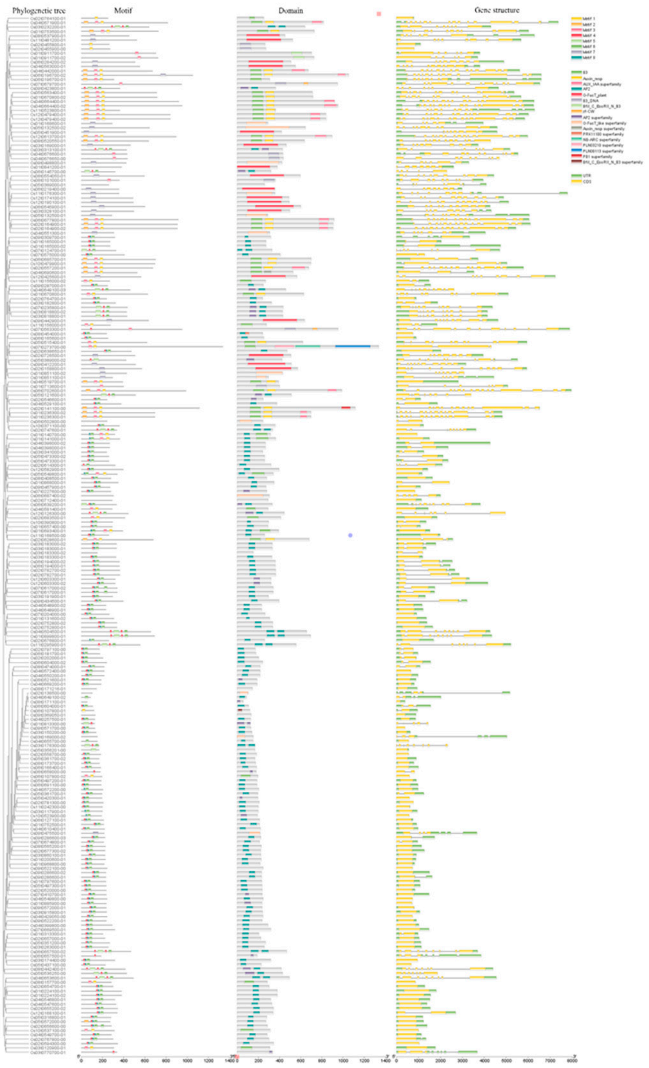

Supplement Figure S1. The analysis of the AP2 gene family is presented in a picture. The far left column shows the Phylogenetic tree and transcript name. The second column represents the motif analysis, while the third column shows the structure domain Analysis and the E-Value < 0.01. The fourth column is highlighted in yellow, which represents the CDS sequence.

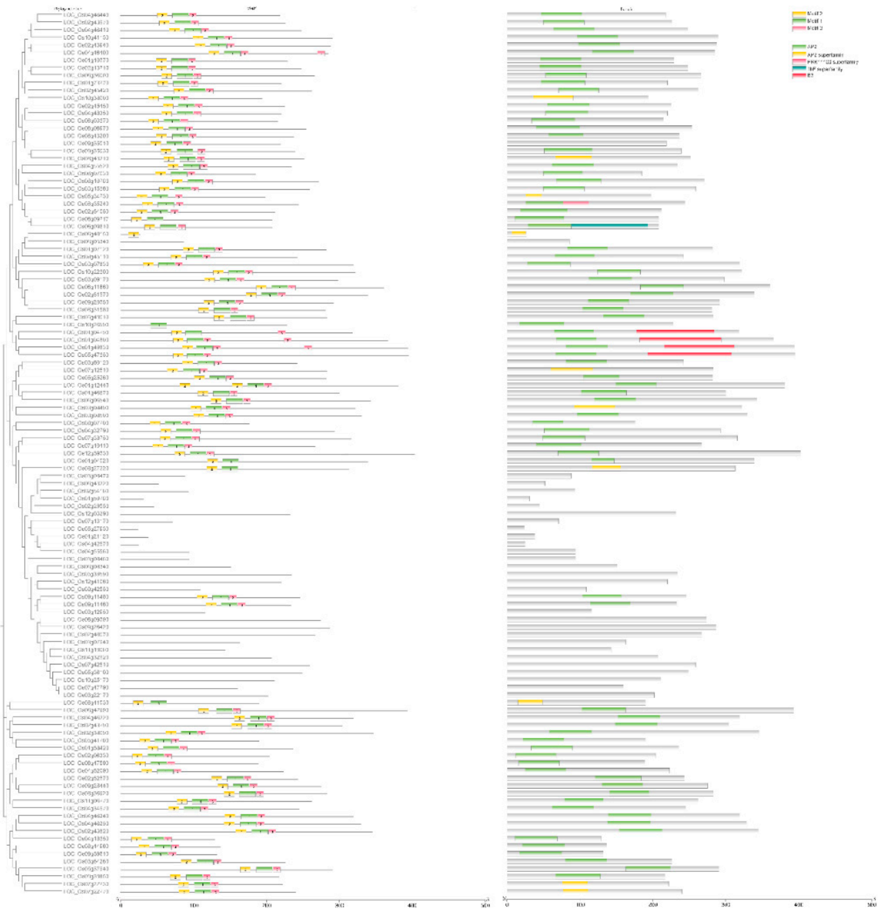

Supplemental Figure S2. The analysis of the gene of transcriptome is presented in a picture. The left column shows the Phylogenetic tree and gene name. The second column represents the motif analysis, while the third column shows the structure domain Analysis and the E-Value < 0.01.
